# Development and Evaluation of the Telehealth in Motor Neuron Disease System: The TIME Study Protocol

**DOI:** 10.2196/57685

**Published:** 2024-10-08

**Authors:** Liam Knox, Elizabeth Coates, Alys Griffiths, Yasmin Ali, Esther Hobson, Christopher McDermott

**Affiliations:** 1 School of Neuroscience University of Sheffield Sheffield United Kingdom

**Keywords:** motor neuron disease, amyotrophic lateral sclerosis, telehealth, digital health, process evaluation, implementation, co-production, digital technology, mhealth, eHealth, virtual medicine

## Abstract

**Background:**

For more responsive care provision for motor neuron disease and caregivers, a digital system called Telehealth in MND-Care (TiM-C) was created. TiM-C sends regular symptom questionnaires to users; their responses are sent to health care professionals (HCPs). To enable people with motor neuron disease to participate in research studies more easily, a parallel platform was developed from TiM-C, called Telehealth in MND-Research (TiM-R). TiM-R can advertise studies, collect data, and make them available to MND researchers.

**Objective:**

This study has 4 work packages (WPs) to facilitate service approval, codevelop the TiM systems, and evaluate the service. Each WP aims to understand (1) what helps and hinders the approval of the TiM-C system as a National Health Service; (2) what aspects of MND care and research are currently unmet and can be addressed through the TiM-C and TiM-R systems; (3) how TiM-C influences MND care, from the perspective of people with motor neuron disease, their caregivers, and HCPs; and (4) the costs and benefits associated with TiM-C.

**Methods:**

WP1 will use semistructured interviews with 10-15 people involved in the approval of TiM-C to understand the barriers and facilitators to governance processes. WP2 will use individual and group interviews with 25-35 users (people with motor neuron disease, caregivers, HCPs, MND researchers, and industry) of TiM-C and TiM-R to understand the current unmet needs of these user groups and how TiM services can be developed to meet these needs. WP3 will use a process evaluation involving 5 elements; local context, engagement, user experiences, service impact, and mechanisms of action. A range of methods, including audits, analysis of routine data, questionnaires, interviews, and observations will be used with people with motor neuron disease, caregivers, and HCPs, both those using the system and those who declined the service when invited. WP4 will use data collected through the process evaluation and known costs to conduct a cost-consequence and budget impact analysis to explore the cost-benefit of the TiM-C service. Most data collected will be qualitative, with thematic and framework analysis used to develop themes from transcripts and observations. Descriptive statistics or *t* tests and chi-square tests will be used to describe and analyze quantitative data.

**Results:**

This study has received ethical approval and has begun recruitment in 1 site. Further, 13 specialist MND centers will adopt TiM-C and the TIME study, beginning in July 2024. The study will conclude in November 2026 and a final report will be produced 3 months after the completion date.

**Conclusions:**

This study will facilitate the implementation and development of TiM-C and TiM-R and fully evaluate the TiM-C service, enabling informed decision-making among health care providers regarding continued involvement and contribute to the wider literature relating to how technology-enabled care services can affect clinical care.

**International Registered Report Identifier (IRRID):**

DERR1-10.2196/57685

## Introduction

Motor neuron disease (MND) is an incurable disease, that causes progressive weakness of muscles involving the limbs, speech, and swallowing, leading to progressive disability and eventual respiratory failure. In the United Kingdom, there are approximately 5000 people with MND at any one time [1]. The average life expectancy following diagnosis is 2 to 3 years but the course of MND can vary from only a few months to over 10 years. The distress and burden of the disease affects people with MND, their family, and caregivers and the relenting progression of disability causes social, emotional, and financial strain [2,3].

In the United Kingdom, multidisciplinary teams (MDTs) deliver specialist MND care through MND Care Centers or care networks across the country. Expert health care professionals (HCPs) monitor symptoms and offer interventions such as Riluzole, which can improve survival by approximately 2 to 3 months [4], gastrostomy feeding to promote good nutrition [4], or treatment of respiratory failure with noninvasive ventilation, which improves both quality of life and life-expectancy [5]. Attendance at specialist MND clinics has been reported to improve survival independent of these other interventions [6,7]. Traditionally, clinics are held at regular fixed intervals, approximately 3 months apart.

Despite the potential benefits of MDT care, this model is not always sufficiently responsive to the needs of patients because these can change rapidly, and therefore, can require people with MND and their families to undertake progressively more difficult journeys to the clinic at a time predicted by the clinician at their last meeting. Given the burden associated with traveling to the clinic, it is important that visits occur when they are most needed. Conversely, some people with MND whose needs may not change considerably over time, may not benefit from a clinic appointment every 3 months.

To supplement traditional care for people with MND and their caregivers, and provide greater information on symptoms for the MDT, the Telehealth in MND-Care on MyPathway system (herein referred to as TiM-C) was codeveloped with people with MND, caregivers, and HCPs. TiM-C is software that enables people with MND and their caregivers to report their progress and symptoms to the hospital MDT from their homes using an app or via the online website [8] (TiM-C). Users are regularly asked to complete a variety of symptom questionnaires (eg, appetite, weight, functional rating, breathing, and mental health), answers to which are securely sent to the clinical portal. An initial evaluation in one center found TiM-C to be easy to use, even for those with significant disabilities, acceptable, and low in burden, taking approximately 5 minutes per week to complete. Around 85% of people with MND would recommend TiM-C to others and 95% would use TiM-C if they could not travel to the clinic. HCPs also found it accurate and easy to use [9,10]. However, additional research is needed to understand how best to implement TiM-C in MND centers, further user needs, the impact of the service, and the costs of the system. TiM-C is currently being adopted as a clinical service within 14 MND specialist centers across the United Kingdom.

**Figure 1:**
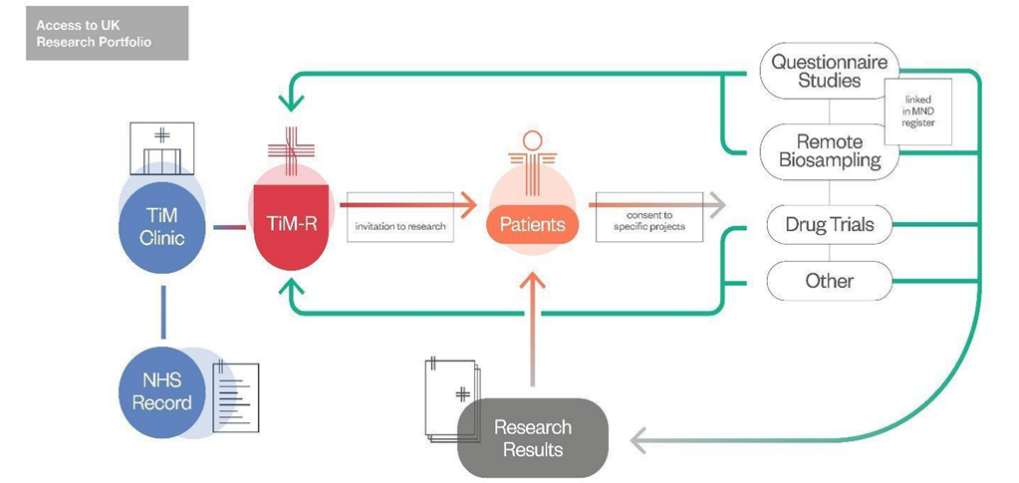
Overview of how TiM-R links patients to research. MND: motor neuron disease; NHS: National Health Service; TiM-R: telehealth in MND-research.

In addition to supporting clinical care, this type of remote monitoring is a useful method for research data collection [11,12]. Therefore, a second system called Telehealth in MND-Research (TiM-R; Figure 1) is being adapted from TiM-C to decrease the burden of MND research and enable people with MND to participate in studies regardless of where they live. However, it is important to codevelop technological systems to best meet the needs of end users [12,13] and research is required to ensure TiM-R is feasible and acceptable for people with MND, caregivers, HCPs, and MND researchers before this is launched in Summer 2024.

The aim of this study is to create an approval strategy alongside information governance experts, further develop TiM-C and TiM-R alongside end users, and understand the effects on clinical practice and cost of the TiM-C clinical service. The following research questions will be answered:

1. From the perspective of individuals involved in the approval of National Health Service (NHS) services, what are the barriers and facilitators of approving TiM-C at scale?
2. What are the unmet needs of people with MND, their caregivers, HCPs, MND researchers, and industry?
3. How can TiM-C and TiM-R be developed to support end user needs?
4. Does the use of the TiM-C service result in better MDT care for people with MND and their caregivers?
5. What are the costs associated with the TiM-C service and how do they affect health care expenditure?

## Methods

### Design

The design of this project was based upon multiple frameworks and guidelines, which have helped develop a logic model, consider outcomes, and data collection methods. These include the MRC framework for the development and evaluation of complex interventions [14], National Institute for Health and Care Excellence Evidence Standards Framework [15], Person-based approach to intervention development [13], and Non-adoption, Abandonment, Scale-up, Spread, and Sustainability (NASSS) [16]. A total of 14 sites will collect data, however, some work packages (WPs) will only use a subset of sites (eg, WP1 only collects data from 5). The project has 4, interconnected WPs, which answer the research questions above:

1. Governance approval strategy development (research question 1)
2. TiM-C and TiM-R codevelopment (research questions 2 and 3)
3. TiM-C process evaluation (research question 4)
4. TiM-C economic evaluation (research question 5)

Through the NASSS framework and the collection of other data in the 2nd and 3rd work packages (described below), we will collect information that can be used to identify barriers that affect TiM-C and TiM-R implementation and use. Throughout the study, using an implementation science lens, we will address identified barriers to facilitate the uptake of the TiM system, using methods suggested by Cane et al [17]. We will document any adaptations made to our governance approval strategy or the TiM intervention.

To reduce the number of under-served groups within this project, the research team will aim to involve a diverse range of participants throughout the entirety of this study and overcome known barriers to participation [18]. This will include remote research activities to reduce the need to travel, support for caregivers to enable participation, and continual data monitoring to ensure representative inclusion of different ages and genders.

Each of the 14 sites involved in this study will be given permission to mildly modify research activities to enable people with different abilities or circumstances, who meet the eligibility criteria, to participate in any and all parts of the study they want to. For example, modifying consent or data collection procedures to enable the participation of people with MND and caregivers who are physically unable to complete documents, or questions being shared ahead of an interview to allow participants who have difficulty communicating time to prepare recorded answers.

### Development of a Governance Approval Strategy (WP1)

This WP will use qualitative research to understand and assess the barriers and facilitators of successful technology approval. The research question in this work package will be addressed using a qualitative approach because there is a dearth of information available on which aspects help and hinder technology governance approval in MND care within the NHS [19]. Therefore, the semistructured interviews with individuals involved in the approval process of the TiM-C system within NHS organizations will provide rich data on this issue [20].

Convenience sampling will be used to recruit 10-15 individuals involved in the approval process of TiM-C (eg, governance, ICT, and data security staff) within 5 NHS organizations (out of the 14 using TiM-C). Semistructured interviews will be conducted using video-conferencing software (in line with participant preferences and permissions) or telephone. The interviews will last approximately 30-60 minutes in duration and be conducted by members of the research team.

The interviews will begin with general, open-ended questions relating to the participant’s experience within their work role, and how they were involved in the approval process of TiM-C. Specific questions will explore what aspects of the approval process went smoothly and what factors they believe contributed to this. Further questions will relate to aspects of the approval process that hindered progress and how these may be overcome in the future. The interview schedule is informed by the NASSS framework, combined with the Complexity Assessment Toolkit (NASSS-CAT) [16] to help identify areas of complexity within NHS organizations.

Interviews will be audio-recorded and transcribed verbatim for analysis. All identifiable information will be removed during transcription with a pseudonym replacing the participant’s name. The research team will conduct a framework analysis using NVivo [21]. The analysis will focus on semantic (as opposed to latent) meanings within the transcripts. The information gained from this WP will be used to inform a toolkit that provides suggestions for future health care interventions aiming to become clinically adopted services within the NHS. This toolkit will be freely available and provide suggestions gained from the governance experts interviewed to decrease the current lengthy process of service adoption.

### TiM-C and TiM-R Development (WP2)

This work package will use a qualitative research design, with semistructured interviews with users of TiM-C and prospective users of TiM-R, including people with MND, caregivers, HCPs, MND researchers, and industry representatives. A qualitative design is most appropriate in order to continue the person-based approach to TiM development [13], and to ensure the intervention remains relevant, acceptable, and feasible, it is important to explore the views of the people who will be interacting with it [22,23]. We will use the information collected throughout this WP to make further refinements to the TiM-C and TiM-R interventions and ensure they meet the needs of end users.

A mixture of individual and group interviews will be used to collect the data, based on participant preference. Purposive sampling will recruit approximately 25-35 participants for this WP (approximately 5-to-7 per user group) across 5 sites. In line with the INCLUDE study [18], methods will be used to recruit those who do not usually engage in research and include a range of demographics (eg, age, gender, and ethnicity), clinical characteristics (eg, duration and severity of MND), and levels of experience with technology to support access to health care and research. We will not set specific numbers we will recruit for each of these categories, but will continually assess our inclusivity throughout recruitment to ensure we are not excluding any group. Interviews will be conducted either face-to-face or using videoconferencing software. The interviews will last approximately 30-60 minutes in duration and be conducted by members of the research team.

All interviews will begin with simple introductory questions to ask the participants about their experience with MND (either having, supporting, or researching MND). Group interviews will also begin with introductions between the various group participants. After the introduction, specific questions will depend on which user group the participant belongs to.

For people with MND and caregivers, questions will relate to what needs they have that are currently not being met and how TiM-C may be developed to meet these needs. Further questions will relate to how they feel about engaging in remote research (eg, consenting, data collection, and building trust through TiM), and any specific developments they believe TiM-R should include.

For HCPs, questions will be asked about how they use TiM-C, elicit unmet needs within their services, what aspects are required to provide good quality care, and how TiM-C could be further developed to help provide these. Questions will also be included relating to the TiM-R development and how this will interact with TiM-C.

For MND researchers and industry, questions will focus on the current difficulties with conducting research within MND, what is required to deliver good-quality research studies, and how TiM-R can incorporate those functions to facilitate the conduct of remote research and large-scale clinical trials.

Interviews will be audio-recorded and transcribed verbatim for analysis. All identifiable information will be removed during transcription with a pseudonym replacing the participant’s name. Participants will have the option to choose their own pseudonyms. The research team will conduct a framework analysis using NVivo [21]. The analysis will focus on semantic (as opposed to latent) meanings within the transcripts. Findings from the analysis will be provided to an intervention development team, consisting of multiple different stakeholders, and used to further develop the TiM-C and TiM-R systems. After developments have been implemented, the intervention development team will then decide whether to conduct an additional round of interviews, using the same methodology described above. These interviews may use the same individuals who participated in the first round.

### TiM-C Process Evaluation (WP3)

This WP will use a mixed methods design to conduct an in-depth process evaluation to explore implementation, mechanisms of action, and context [24], and investigate the impact the TiM-C system has on MND specialist center’s services. Purposive sampling will be used to recruit people with MND, their caregivers, and HCPs using TiM-C to participate in semistructured interviews, case studies, and quantitative surveys (Multimedia Appendix 1 presents an overview of when these activities will be conducted).

A process evaluation will be conducted because it is not feasible to evaluate a complex digital intervention such as TiM-C via a randomized controlled trial [10,24]. There are 5 components in this process evaluation, “local context,” “engagement,” “user experiences,” “service impact,” and “mechanisms of action”, each with its own section below and in Multimedia Appendix 1. Data collected on these components will enable an assessment of context, implementation, and mechanisms of action [24]. A triangulation protocol adapted from Farmer et al [25] will be employed to separately conduct and analyze each component before triangulating the findings.

### Description of Service and Local Context

To collect information regarding how the local context may affect the delivery and outcomes of the TiM-C service, sites will complete a survey after they begin using the system. This survey will include questions relating to what HCPs are included in the local MDT, who reviews the TiM system and how often, how issues are escalated or referred to other HCPs, and what complexities exist within the organization (informed by the complexity assessment tool of the NASSS).

Additionally, most MND MDT services conduct biannual audits. Where available, the last 2 audits will be collected to provide an overview of the level to which each site meets National Institute for Health and Care Excellence guidelines.

### Engagement

Manually and automatically collected data at every site will be analyzed in accordance with an adapted version of the taxonomy of adherence Vrijens et al [26]. This will provide information relating to the uptake, initiation and discontinuation, persistence, nonpersistence, and activity of the TiM-C service.

Local HCPs will keep a record of the number of people with MND who have been invited to TiM-C and the reasons for people not creating an account if these are offered at the time of face-to-face invitation. The TiM-C system automatically collects the number of people with MND who create an account. The TiM-C system also automatically collects the number of caregivers who are invited and the number who create an account. Combining this data will provide information on uptake (the proportion of people with MND and caregivers who create an account when invited) at each site, in addition to reasons for nonuptake that can be used to develop the invitation process.

If available at the MND center, demographics and routinely collected data will be analyzed to investigate whether there are any statistically significant differences between those who do and do not create TiM-C accounts. Relevant data may include, age, gender, partial postcode (to explore the effects of deprivation), and outcomes relevant to MND such as functional rating, date of onset, site of onset, and date of diagnosis. Statistical analyses will include *t* tests and chi-square tests for parametric and nonparametric data, respectively.

The TiM-C system automatically collects anonymized data relating to the date and time people with MND and caregivers complete a questionnaire. This data will be analyzed to calculate initiation and discontinuation (the date of first and last questionnaire completion, respectively), persistence (the time from first to last questionnaire completion), nonpersistence (the time following persistence during which no questionnaires are completed), and activity (the percentage of questionnaires completed during persistence). Similar to the above, if data is available, statistical analyses will be conducted to investigate whether any aspect of engagement can be predicted by individuals’ demographics or disease-relevant data.

TiM-C also automatically collects the number of times an HCP logs into the system and sends feedback or resources to people with MND and caregivers. These data will be aggregated to calculate how often the TiM-C system is reviewed and used to send information to people with MND and caregivers.

### People With MND, Their Caregivers, and HCP (User) Experiences

All TiM-C users will be invited to complete a short questionnaire to assess the acceptability and feasibility of the system, 1 month and 12 months after they create their TiM-C accounts. Questions will be based on the Theoretical Framework of Acceptability [27].

Additionally, 10 semistructured interviews will be conducted with people with MND, their caregivers, and HCPs at 2 time points (60 in total) from up to 5 centers, to facilitate understanding of the experiences, perceived impacts on knowledge, and access to care, and potential unintended consequences of the TiM-C system. Interviews will be conducted after users have had their TiM-C accounts for 3 months and 12 months.

Interviews will use videoconferencing software (in line with participant preferences and permissions) or telephone. All interviews will begin with general, open-ended questions relating to the participant’s understanding of MND and their perception of the care they receive, or the participant’s role and experience with MND care. For people with MND and their caregivers, specific questions will then relate to their experiences of the TiM-C service and how this has affected their care. For HCPs, specific questions will relate to their understanding of MND in general and of the people with MND and caregivers using TiM-C. Further questions will be asked about how HCP’s use the TiM-C system and their perception of how it affects the care that they deliver.

The interviews will last approximately 30-60 minutes in duration and be conducted by members of the research team. Interviews will be audio-recorded and transcribed verbatim for analysis. All identifiable information will be removed during transcription with a pseudonym replacing the participant’s name. Participants will have the option to choose their own pseudonyms. An inductive reflexive thematic analysis [28] will be conducted by the research team using NVivo. The analysis will focus on semantic (as opposed to latent) meanings within the transcripts.

### Service Impact of TiM-C Upon MDT Care and the MND Service

To explore how TiM-C has affected the structure of each organization’s MDT care, the same survey completed to describe the service and local context (see “TiM-C process evaluation” section above) will be completed yearly after the first. Additionally, any audit on the MND MDT services conducted within the duration of this study will also be collected.

Semistructured interviews will be conducted with HCPs using the TiM-C system to explore their beliefs about their connectedness with users, timeliness of care, and the quality of care that they deliver. These questions will be asked at the same time as those described in 4.3.3 above, to reduce the burden the research has on participants.

To explore whether the TiM-C system has improved the experience of MND care for people with MND and their caregivers, participants will complete a patient-reported experience measure (PREM) 1 month and 12 months after they have been invited to create TiM-C accounts. Participants who have created an account will be allocated to the “user” group and those who do not create accounts will be allocated to the “non-user” group. The PREMs will be compared between these 2 groups across both data collection points. The PREM is adapted from the Client Satisfaction Scale [29].

### TiM-C Mechanisms of Action

In-depth case studies will be used to explore how HCPs use the data from TiM-C during clinical appointments and MDT meetings, which will enable an investigation into TiM-C’s mechanisms of action. Yin [30] suggests that 5 case studies are appropriate for complex interactions and 3 for literal replications. Therefore, a collective case study approach will be adopted using 5 MND centers. Within each case study, data will be collected relating to how TiM-C is used during 3 clinical appointments and 3 MDT meetings.

A combination of data collection techniques will be used, including focused ethnography, consisting of observations and field notes within clinical appointments and MDT meetings, alongside short questionnaires, and related document analysis. Information from the other elements of this process evaluation will also be incorporated into the case studies (eg, engagement data).

Members of the research team will conduct direct, nonparticipant observations of 3 clinical appointments and 3 MDT meetings. The same researcher will take field notes and potentially ask follow-up questions after the appointment or MDT meeting has finished. The researcher will either attend such activities physically or join virtual meetings. Appointments and MDT meetings will be audio-recorded and transcribed verbatim for analysis. Framework analysis will be used to analyze the transcripts and field notes.

A short, bespoke questionnaire was created to explore how HCPs perceived the use of the TiM-C data during MDT meetings. The questionnaire includes items on the trustworthiness of TiM-C data and whether and how the HCP acts on the data. All HCPs attending the MDT will be asked to complete this questionnaire after the meeting or appointment has finished.

Local HCPs and researchers will collect other documents relating to the use of TiM-C for the people with MND discussed at the clinical appointments or MDTs (eg, TiM-C messaging data, clinical notes, and emails) and securely transfer them to the central research team for analysis. Only data relating to TiM-C will be used. This information is necessary to identify how TiM-C data is used to support people with MND and caregivers outside of meetings. Farmer et al [25] triangulation protocol will be used to analyze the data set, due to the large amount of data involved, collected from multiple sources. Each case study will be analyzed separately before similarities and differences are considered in cross-case comparisons.

### TiM-C Economic Evaluation (WP4)

This WP includes a health economic analysis of the TiM-C system, as it is implemented in MND specialist centers and networks across the United Kingdom. A cost-consequence analysis [31] will form the primary economic evaluation because TiM-C is a complex digital intervention with multiple possible effects, including both health and nonhealth outcomes, which makes the creation of a single measure difficult. A budget impact analysis [32] will also be conducted, to help future health care commissioners understand how the implementation of TiM-C may affect expenditure under different circumstances.

The cost-consequence analysis will only focus on costs for intervention delivery, as TiM-C is not designed to replace any part of usual care (ie, TiM-C will incur costs in addition to usual care, rather than accrue cost-savings). Per patient and caregiver costs incurred from the need for a HCP to regularly review user scores, will be calculated by asking HCPs to estimate how long it takes to review the account of a single user. This will be combined with unit costs of health, accessed from the Personal Social Services Research Unit. These will be combined with the central cost of TiM-C per MND service, which is already known, in addition to HCP time to receive the necessary training. Outcomes will be taken from the process evaluation and presented alongside the per-user cost of TiM-C to aid comparison.

A budget impact analysis will be conducted using the costs calculated as part of the cost-consequence analysis described above. Engagement data from the process evaluation will then be used to describe the cost of TiM-C for the MND center (in terms of HCP time and system cost) under different review protocols. For example, TiM-C does not dictate how often HCPs should review the system. The more often TiM-C is reviewed and the higher the user engagement from people with MND and caregivers, the more HCP time will be needed and the higher the associated costs. This analysis will display these different circumstances to aid health care commissioners in understanding the affordability of the TiM-C service.

### Participants

Participant numbers are provided within each WP and each section of the process evaluation. Where numbers are not provided, we will recruit everyone who meets the below eligible criteria (Textbox 1; eg, The PREM described in the section “Service impact of TiM-C upon MDT care” and the MND service will be sent to every individual with MND at each site). To be as inclusive as possible, there are no exclusion criteria.

**Eligibility criteria for each participant group.**

Patient inclusion criteria:

- Aged 18 years or older
- Diagnosis of clinically definite, lab supported, clinically probable, or possible motor neuron disease (MND) by the El-Escorial criteria and additionally the Progressive Muscular Atrophy variant where appropriate investigation has excluded mimics of MND
- Capacity to give informed consent
- Has been invited to create a TiM-Care (TiM-C) account at least 1 month ago
- Can understand written/verbal English

Caregiver inclusion criteria:

- Aged 18 years or older
- Capacity to give informed consent
- Has been invited to create a TiM-C account at least 1 month ago
- Can understand written/verbal English

Health care professional inclusion criteria:

- Health care professionals (HCPs) who provide support to people with MND, including medics, specialist nurses, dietitians, speech and language therapists, physiotherapists, occupational therapists, health care assistants, etc.
- Part of an MDT that has used the TiM-C system for at least 1 month
- Can understand written/verbal English

MND research inclusion criteria:

- Aged 18 years or older
- Any researcher who has previously or is currently conducting research with people with MND, their caregivers, or HCPs.
- Can understand written/verbal English

Industry staff inclusion criteria:

- Aged 18 years or older
- Currently or recently (within last year) used by an industry organization that has conducted clinical research involving people with MND. This includes clinical trials, questionnaire-based studies, qualitative research, or telehealth research.
- Can understand written/verbal English

### Consent

All potential participants to each WP will be provided with written information about the nature and objectives of the study in the form of a Participant Information Sheet and given a minimum of 24 hours to decide whether to take part. However, a participant can consent immediately if they wish. Potential participants will also be given the opportunity to discuss the research with the relevant researcher and ask questions either in person or via contact details supplied in the Participant Information Sheet. Potential participants will be reminded that their participation is entirely voluntary and that they have the right to withdraw at any point (without it affecting the service provision for people with MND). Formal written consent will be obtained for all research participants, the process for which will be adapted for those participants unable to physically provide this. Consent may be taken remotely by verbally recording the participant, only if necessary. The consent procedure will be completed by the researcher/interviewer prior to any interview or observation. They will explain the study and make sure each person fully understands what they are agreeing to.

For the quantitative elements of the study, participants can immediately complete any questionnaires they have consented to. For the qualitative elements of the study, the local teams will pass on their contact details to the central research team. The research team will then contact the potential participants to provide them with more information about the study. Where relevant, the next step will be for the researcher to organize a mutually convenient time and place for the interview. Participants will not be offered payment for taking part in this study. Should interviews be conducted face-to-face, reasonable travel expenses will be reimbursed for participants.

Within the MDT observations, there is the possibility that some HCPs consent to be observed, whereas other HCPs do not provide this consent. In such a situation, the observation will still continue and be recorded. However, all instances where a nonconsenting HCP speaks will be removed from the transcript and any identifiable information about nonconsenting HCPs, such as names or job titles, will be removed. Nonconsenting HCPs will not be asked to complete a questionnaire.

### Nonexplicit Consent

Within the engagement section of the TiM-C process evaluation, this study proposes to analyze data on people with MND and their caregivers, which has been collected as part of running the MND clinical service. This data will only be used to investigate engagement with TiM-C and facilitate the exploration of reasons for the engagement and nonengagement with the TiM-C service.

As this data is collected as part of the standard running of the MND service, explicit consent will not be sought from people with MND and their caregivers before this data is analyzed. All individuals who have opted out via the national data opt-out service will have their data removed from this analysis. The legal basis for the use of this data is to improve or benefit health and care by monitoring the use of this novel intervention to investigate how effective it is.

### Ethical Considerations

This study was approved by the Health Research Authority and NHS Research Ethics Committee (reference number: 22/NI/0192). Informed consent is (or waiver thereof) and the process used to obtain this, is fully described in the section above. All data are anonymized, with participants being given a number (quantitative) or pseudonym (qualitative). Participants were given the choice of their own pseudonyms. No compensation or remuneration was provided as an incentive for any WP described within this protocol. However, reimbursement for reasonable expenses incurred (eg, travel to interviews) was provided to participants.

## Results

The study received ethical approval in January 2023 (22/NI/0192) and is currently being set up in MND specialist centers in the United Kingdom. Thirteen MND researchers and industry representatives have been recruited to the TiM-C and TiM-R development (WP2). Framework analysis has identified the current unmet needs of these participant groups and the functionality that can be implemented into TiM-R. This work will be presented at the MND Association 2024 symposium and published shortly.

## Discussion

### Principal Findings

This study will collect data relating to NHS clinical approvals, TiM-C and TiM-R development, and evaluate the TiM-C service. The project will open approximately 14 sites to recruit to all WPs. The study is due to be completed in November 2026, and the final report will be produced within 3 months of the study closing. However, we will also separately disseminate the results for each WP. We anticipate that the data from WP1 will enable the development of a toolkit to aid the approval process of future health care interventions in the NHS, which will be disseminated through a publication and publicly available website. This responds to our group’s collaboration with multiple industry partners who describe significant delays in governance approval processes. Findings from WP2 will primarily inform the refinement of TiM-C and TiM-R, but could also be useful for future researchers who wish to develop innovative digital services for people with long-term conditions. We will publish this through 2 academic publications.

WP3 and WP4 will collect data relating to the current provision of MND services and the effect of TiM-C on this provision. We anticipate that this will enable greater conclusions to be drawn relating to the benefits and consequences of the TiM-C service, whilst also enabling the testing of our program theory and logic model. As per the MRC’s guidelines for complex intervention development [14], we will explore whether to further explore implementation and effectiveness depending on the results of the process evaluation. Alongside stakeholders, we will publish this work academically but also work with wider groups (eg, NHS decision makers, policy groups, and charities) to increase the impact of this work.

### Limitations

A limitation of this study is the exclusion of a randomized controlled trial design, which is widely considered to provide one of the highest levels of evidence for or against an intervention. The decision to focus primarily on a process evaluation was taken due to the guidance provided by the MRC [14], which acknowledges that randomized controlled trials are not suitable in all situations, and a previous feasibility study indicating that such a design would not be feasible [10]. Nevertheless, a future study could reexamine the feasibility of conducting a randomized controlled trial and, if possible, implement this design to provide the best quality evidence relating to the effectiveness of TiM-C.

### Conclusions

This study will collect and publish information to support the efficient approvals of remote monitoring systems in the NHS (WP1), further coproduce the TiM-C and TiM-R services (WP2), and fully evaluate the TiM-C service (WP3 and 4). This work will enable health care decision makers to make an informed decision regarding whether to continue using the TiM services within their specialist centers and to support research.

## Appendix

Multimedia Appendix 1 A visualization of when each activity will be conducted. All timings are ±1 month.

## Abbreviations

CAT: Complexity Assessment Toolkit
HCP: health care professional
MDT: multidisciplinary team
MND: motor neuron disease
NASSS: Non-adoption, Abandonment, Scale-up, Spread, and Sustainability framework
NHS: National Health Service
PREM: patient-reported experience measure
TiM: telehealth in motor neuron disease
TiM-C: TiM-Care
TiM-R: TiM-Research
WP: work package

## Data Availability

The data sets generated during and analyzed during this study are available from the corresponding author on reasonable request.
