# Supplementary material for: Development and Evaluation of the Telehealth in Motor Neuron Disease System: The TIME Study Protocol
Source: JMIR Res Protoc. 2024 Oct 8;13:e57685. doi: 10.2196/57685 (PMC11496908; doi:10.2196/57685)
Supplement: Multimedia Appendix 1 [file resprot_v13i1e57685_app1.docx]

**Table S1.** A visualization of when each activity will be conducted. All timings are ±1 month.

| Research activity | | Baseline | Month 1 | Month 3 | Month 12 |
| --- | --- | --- | --- | --- | --- |
| **Local context** | | | | | |
|  | Surveys to gather information regarding local set up and complexities | ✓ |  |  | ✓ (yearly) |
|  | MNDA audits | The last 2 completed and any conducted during the study | | | |
| **Engagement** | | | | | |
|  | Engagement data | Longitudinally. Analyzed yearly. | | | |
| **User experiences** | | | | | |
|  | Acceptability questionnaire (everyone) |  | ✓ |  | ✓ |
|  | 60 semistructured interviews with people with motor neuron disease, caregivers, and HCPs^a^ |  |  | ✓ | ✓ |
| **Service impact** | | | | | |
|  | 20 HCP interviews (incorporated into user experience interviews) |  |  | ✓ | ✓ |
|  | Patient reported experience measure |  | ✓ |  | ✓ |
| **Mechanisms of action (case studies)** | | | | | |
|  | Observations of 30 people with motor neuron disease (MDT^b^ meetings and appointments) with associated field notes | According to arrangement, can be across entire study | | | |
|  | Short HCP questionnaires | After an observed MDT | | | |
|  | 30 sets of document analysis | Any TiM-C^c^ related documents for observed people with motor neuron disease | | | |

^a^HCP: health care professional.

^b^MDT: multidisciplinary team.

^c^TiM-C: TiM-Care.
